# Differential-display reverse transcription-PCR (DDRT-PCR): a new technology for molecular detection and studying one of the antagonistic factors of *Bacillus endophyticus* strain SA against *Staphylococcus aureus* (MRSA)

**DOI:** 10.1007/s13205-016-0439-1

**Published:** 2016-06-03

**Authors:** Mahmoud F. Moustafa, Tarek H. Taha, M. Helal, Sulaiman A. Alrumman

**Affiliations:** 1 Department of Biology, College of Science, King Khalid University, Abha, Saudi Arabia; 2 Department of Botany, Faculty of Science, South Valley University, Qena, Egypt; 3 Environmental Biotechnology Department, GEBRI Institute, City of Scientific Research and Technology Applications, New Borg El-Arab City, Alexandria, Egypt; 4 Department of Biology, Faculty of Science and Arts, Northern Borders University (Rafha), Arar, Saudi Arabia; 5 Botany and Microbiology Department, Faculty of Science, Al-Azhar University, Cairo, Egypt

**Keywords:** Endophytic, *Bacillus endophyticus*, Methicillin resistant *Staphylococcus aureus*, GC–MS, Differential display RT-PCR

## Abstract

Differential Display (DDRT-PCR) is a powerful technique for analyzing differences in gene expression. In-vivo expression technologies and differential display RT-PCR are providing new approaches to further examine a microbe’s response to experimental conditions which more closely resemble natural microbial associations and habitats. In this study, *Bacillus endophyticus* strain SA isolated from the inner tissue of the stem of the cultivated plant (*Salvadora persica*, Asir, Kingdom of Saudi Arabia) produces an antagonistic factor. This factor has a broad spectrum of activity against Gram-positive and specifically against *Staphylococcus aureus* (MRSA). The antagonistic factor was isolated from the bacterial culture medium and purified by thin layer chromatography technique, then analyzed by GC–MS analysis. Identification of the producer strain was performed using the partial nucleotide sequence of 16S rRNA gene, which indicated that this strain is identical to *B. endophyticus* with 99 % similarity. The sequence of this strain was deposited at NCBI GenBank under accession number KF011545. Application of differential display RT-PCR revealed that the isolate was able to up-regulate a gene with serine protease like protein. The protein is well known as antimicrobial agent and was reported to be produced by plants, animals and insects. Serine protease is also known to be produced by bacteria for purposes oth er than bacterial–bacterial antagonistic effect, which has been confirmed by this study.

## Introduction

Endophytes have been defined as microorganisms (bacteria or fungi) which for all or part of their life cycle reside within the inner parts of plant tissues, but cause no symptoms of disease (Vega et al. 2005; Khan and Doty 2009). They play an important role in the plant health preservation in addition to their ability to supplement their host plants with inorganic nutrients via nitrogen fixation and iron solubilization processes (Porras-Soriano et al. 2009). In addition, these microorganisms play an important role to protect their host plants from phytopathogenic fungi and bacteria (Emmert and Handelsman 1999). They are able to antagonize the pathogenic microbes through the competition for space and nutrients, production of antibiotics, production of hydrolytic enzymes and by inducing plant defense mechanisms (Ryan et al. 2008; Strobel and Long 1998; Wipat and Harwood 1998). The production of antibiotics and hydrolytic enzymes is a feature of many endophytic bacilli, including *Bacillus* *cereus* (Pleban et al. 1997), *Bacillus pumilus* (Pleban and Sørensen 1996) and *Bacillus subtilis* (Sharga and Lyon 1998).

It has been reported that many endophytic isolates provide beneficial effects to their hosts, like preventing disease development by synthesizing novel compounds and antifungal metabolites (Khan and Doty 2009). Exploring these novel compounds and metabolites may lead to the discovery of new drugs for antibiotic resistant pathogenic bacteria like methicillin resistant *Staphylococcus aureus* (MRSA).

This study focuses on the isolation of endophytic bacteria which are able to cease and inhibit the growth and spreading of methicillin resistant *S. aureus* strain. It also tries to understand the mechanism of action using both of chromatographic and molecular levels.

## Materials and methods

### Plant materials

*Salvadora persica* L. (Arak, *Galenia asiatica*, Meswak, Peelu, Pīlu, *Salvadora indica*, toothbrush tree or mustard tree) stem parts were collected from Asir region, Kingdom of Saudi Arabia and were cut into 10 × 10 mm cubic pieces and used as the essential parts for the isolation of endophytic bacterial isolates.

### Isolation of endophytic bacteria

Miswak stem parts were surface sterilized using three successive washes of 70 % ethanol for 5 min, 1.5 % sodium hypochlorite for 15 min and three rinses with distilled water over a period of 20 min each. The sterilized cubic stem parts were then divided into two pieces using a sterilized razor blade. The inner parts of the stem tissues were placed on nutrient agar plates (OXOID™, United States) and incubated at 30 °C for 3 days. The cultivated endophytic bacterial isolates were transferred to new nutrient agar (NA) plates for purification. The grown separate pure colonies of bacterial isolate were tested for their antagonistic activity against *Klebsiella pneumoniae*, *Escherichia coli* and methicillin resistant *S. aureus* (MRSA).

### Antagonistic effect of SA isolate against *S. aureus* (MRSA)

The antibacterial activity of the chosen bacterial isolate, named SA, was examined against *S. aureus,* according to (He et al. 2009) with some modifications. Both of the pathogenic strain *S. aureus* and the endophytic isolate (SA) were separately cultivated in 20 ml nutrient broth (OXOID™, United States) and incubated at 30 °C at 200 rpm for 24 h. After the incubation period, the *S. aureus* strain was spread over nutrient agar plates using sterile cotton swab. On the other hand, approximately 10^6^ cfu/ml of the isolate (SA) broth was loaded to sterile filter paper discs and followed by addition to the surface of the pathogen spread plates. The plates were finally incubated at 30 °C for 24 h, where the diameter of the clear zones was measured and recorded.

### Comparing the antagonistic effect of the isolated bacteria (SA) with different antibiotics

To rate and determine the exact effect of the isolate SA against *S. aureus* strain, comparison between the SA isolate and different antibiotics was achieved using disc diffusion method technique. Both of sterile filter paper discs were loaded with the isolate SA and ten different antibiotic discs. These antibiotics, namely Metronidazole, Cefotaxime, Cefazolin, Chloramphenicol, Sulphamathoxazole, Cefadroxil, Clarithromycin, Clindamycin, Roxithromycin and Cefoxitin with concentration of 30 µg each, were tested against *S. aureus* spread NA plates. After 24 h of incubation at 30 °C, the diameter of the resulted inhibition zones was measured and recorded.

### Extraction of the bioactive metabolites produced by SA isolate

The extraction of the active metabolites produced by the endophytic isolate SA was done as follows: the surfaces of ten NA plates were spread by *S. aureus* strain, where approximately 10^9^ cfu/ml of the SA isolate broth was added at five different positions to each plate. The plates were incubated at 30 °C for 24 h, where the clear zones between the two kinds of bacteria at which there is no bacterial growth were cut and removed using a sterile razor blade. The collected agar pieces (clear zones) were assumed to contain the active materials, and were extracted using absolute ethanol. The collected agar pieces were added to glass bottle contains 100 ml absolute ethanol, grinded into very small pieces using sterile spatula and were then kept at 4 °C for 1 week. The mixture was filtered using filter papers to remove the agar medium pieces and the ethanolic filtrate was undergoing to GC–MS analysis as described previously (Ezhilan and Neelamegam 2012; Moustafa et al. 2013). GC–MS mass spectrum was explicated using information in the National Institute Standard and Technology (NIST) to know unidentified chemicals.

### Molecular identification of the endophytic isolate

#### DNA extraction

Genomic DNA of SA isolate was extracted according to the instruction manual of DNA extraction kit (Qiagen, Germany).

#### Amplification and sequencing of the 16S rRNA gene

The 16S rRNA gene was amplified using the Multiplex PCR Kit (Qiagen, Germany) with specific universal primers. The sequence of the forward primer was 5′-AGAGTTTGATCMTGGCTCAG-3′ and the sequence of the reverse primer was 5′-AAGGAGGTGWTCCARCC-3′. The PCR mixture consisted of 10 pmol of each primer, 10 ng of chromosomal DNA, 200 μM dNTPs and 2.5 units of Taq polymerase with 10 μl of polymerase buffer containing MgCl_2_. The PCR was carried out starting with one cycle at 94 °C for 10 min followed by 35 cycles of 94 °C for 1 min, 57 °C for 1 min and 72 °C for 2 min followed by final extension step at 72 °C for 10 min. After completion, a fraction of the PCR mixture was examined using 1.5 % agarose gel in TBE buffer (pH 8.5). Electrophoresis was carried out for 30 min at 150 V and the PCR product was visualized using gel documentation system (GelDoc 2000). The PCR product was used directly for purification and sequencing (Macrogen Inc., Korea). The obtained sequence was compared with the sequences deposited in GenBank data base (http://www.ncbi.nlm.nih.gov/) and the alignments between the sequences and the phylogenetic tree were performed using MEGA 5, software.

#### Molecular detection of the antagonistic factor using differential display RT-PCR technique

To investigate the upregulated or downregulated genes during the antagonistic process, both of the pathogenic and the antagonistic bacteria were grown either separately on two different nutrient agar plates or close to each other on the same plate.

#### RNA extraction

The two bacterial strains (the endophytic isolate SA and *S. aureus*) either grown separately or together were submitted to total RNA extraction according to the instructions of the used RNA extraction kit (Qiagen, Germany). The successful extraction of RNA was confirmed using 1 % agarose gel electrophoresis for 30 min at 120 V.

#### Reverse transcriptase PCR (cDNA synthesis)

At first, 5 μl of total RNA were treated with DNase1 enzyme (Invitrogen, USA) to emphasize complete absence of the genomic DNA. The RNA was eluted with 100 μl of an elution buffer followed by incubation with 70 units of DNase1 at 37 °C for 1 h. The enzyme was then inactivated at 85 °C for 1 h and the RNA was precipitated using 100 μl of isopropanol followed by centrifugation at maximum speed (13.000 rpm) for 30 min at 4 °C. Two 70 % ethanol washes were followed, using the same centrifugation settings. The ethanol was removed and the RNA pellets were allowed to air dry for 15 min, at which point, 30 μL RNase-Free water were added. The mRNA was then transcribed to cDNA using a TaqMan Reverse Transcriptase kit (Applied Biosystems) at the presence of Ea1: TTTTATCCAGC and Ea2: ACTTTACGCAG primers in two separate reactions according to the manufacturer’s instructions. The mixture was then incubated at 37 °C for 1 h followed by enzyme inactivation step at 85 °C for 10 min.

### Differential display PCR (DD-PCR)

A total reaction volume of 25 μl containing 2.5 μl 10X Taq buffer, 2.5 μl MgCl_2_, 2.5 μl dNTPs, 1 U Taq DNA polymerase, 3 μl of 10 pmol primer OP1 5′-ACGGQACCTG-3′, 2 μl of each cDNA and finally 12 μl of sterile dH_2_O were mixed. The amplification program was as follows; one cycle at 94 °C for 5 min (hot start), followed by 40 cycles at 94 °C for 1 min, 35 °C for 1 min and 72 °C for 1 min and a final extension step at 72 °C for 7 min. PCR products were visualized on 2 % agarose gel and photographed using gel documentation system (Gel Doc 2000). The up or down regulated genes were excised from the gel and submitted for purification using a gel purification kit (Qiagene, Germany) and the purified PCR products were submitted for sequencing (Macrogen Inc., Korea). The obtained sequences were compared with the sequences deposited in GenBank data base (http://www.ncbi.nlm.nih.gov/).

## Results and discussion

### Isolation and antimicrobial activity of endophytic bacteria

**Fig. 1:**
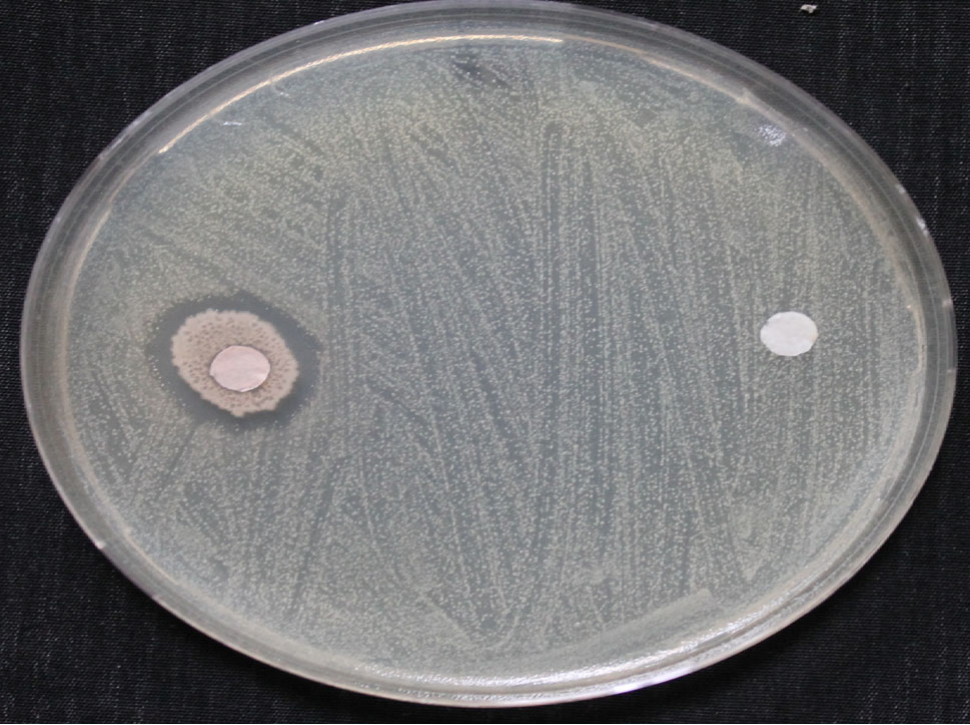
Antagonistic effect of endophytic isolate (SA) against *S. aureus* (MRSA) on a nutrient agar plate

It is well known that, the isolation of bacteria that are capable of invading the inner tissues of a certain plant is a critical process. Plant surface sterilization is necessary to avoid the co-contamination with superficial bacteria. Using of ethanol and sodium hypochlorite at certain dilutions is recommended for complete removal of the plant surface bacteria (Jan et al. 2013). Miswak stem parts were surface sterilized followed by the isolation of inner bacterial isolates on NA plates. About ten bacterial colonies with the same colony shape and characteristics were obtained. One of the colonies was chosen and transferred to a new NA plate to ensure complete purification. The purified bacterium was checked for its ability to antagonize some human pathogenic bacteria including *Klebsiella pneumoniae*, *Escherichia coli* and *S. aureus* (MRSA). Sterile filter papers loaded by approximately 10^6^ cfu/ml of the isolate broth were posted to the surface of pathogens spread plates. The antagonistic effect was only observed with *S. aureus* (MRSA) (Fig. 1), where 2 cm diameter clear zone was recorded.

### Impact of isolate (SA) activity in comparison with different antibiotics

**Fig. 2:**
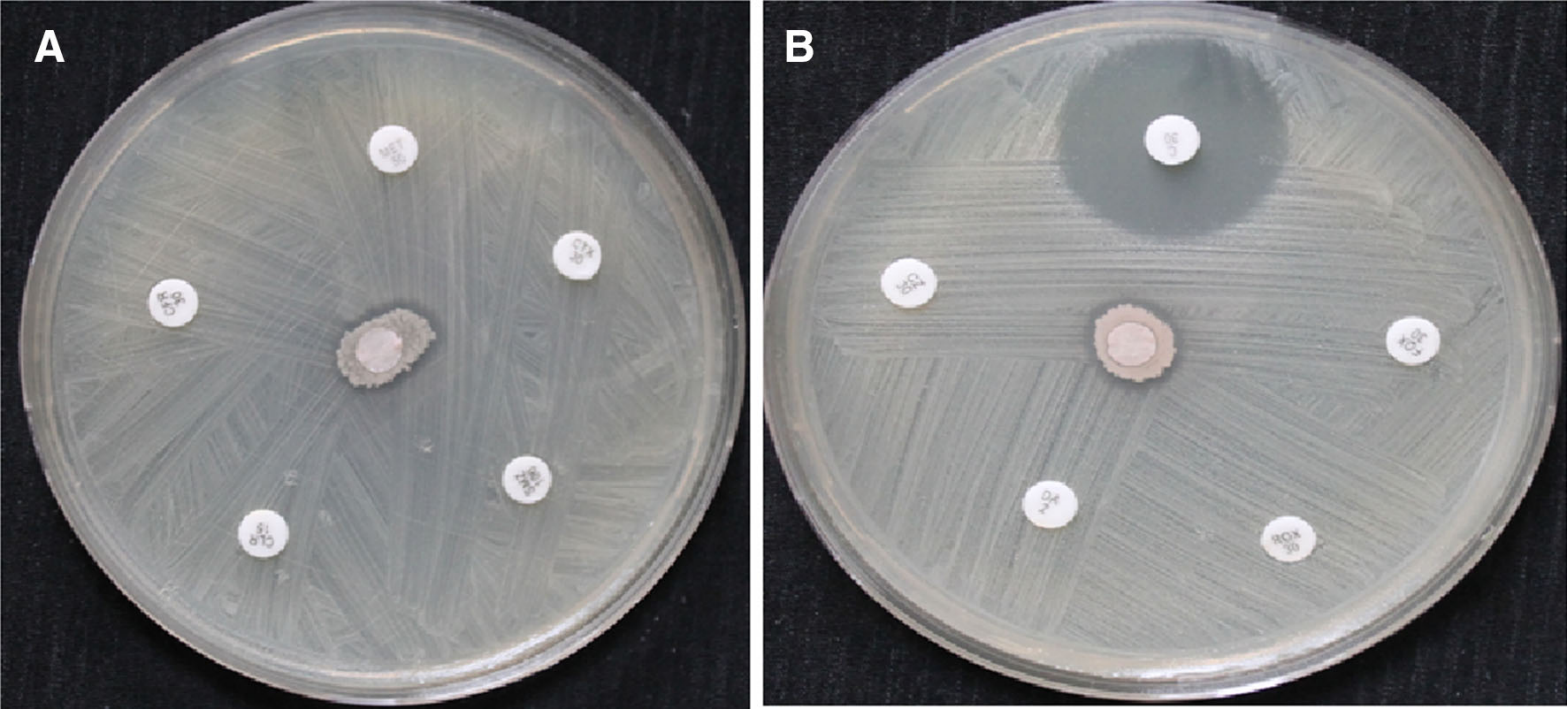
Antibacterial effects of antibiotics compared with endophytic isolate SA against *S. aureus* (MRSA), **a** Cefadroxil, Clarithromycin, Metronidazole, Cefotaxime and Sulphamathoxazole compared with Endophytic isolate, **b** Cefazolin, Chloramphenicol, Cefoxitin, Roxithromycin and Clindamycin compared with Endophytic isolate

The antagonistic activity of the endophytic bacterial isolate was compared with ten different known bacterial antibiotics against *S. aureus* (MRSA). It was observed that 9 of the tested antibiotics hadn’t revealed any kind of antibacterial activity compared with 1.2 cm clear zone recorded by the endophytic isolate (Fig. 2a, b). These results confirm the common concept regarding the ability of microorganism to develop new strategies to resist more and more antibiotics, which invite the researchers to find new and effective antimicrobial agents. On the other hand, only chloramphenicol recorded 2.3 cm clear zone that seems to be two times higher than that recorded with our isolate (Fig. 2b). Although chloramphenicol showed higher activity than our endophytic isolate; the antagonistic activity of this isolate is more preferred than this antibiotic. Later treatment of MRSA strains could be achieved using the bioactive components produced by our endophytic isolate with prohibition of antibiotics especially chloramphenicol. The reasons for this hypothesis came from that there are many recorded disadvantages of chloramphenicol including the risk of its side effects, rising of resistance rates and uncomfortable posology (Diniz-Santos et al. 2006).

### Components in the ethanol extract of inhibition zone slices

**Fig. 3:**
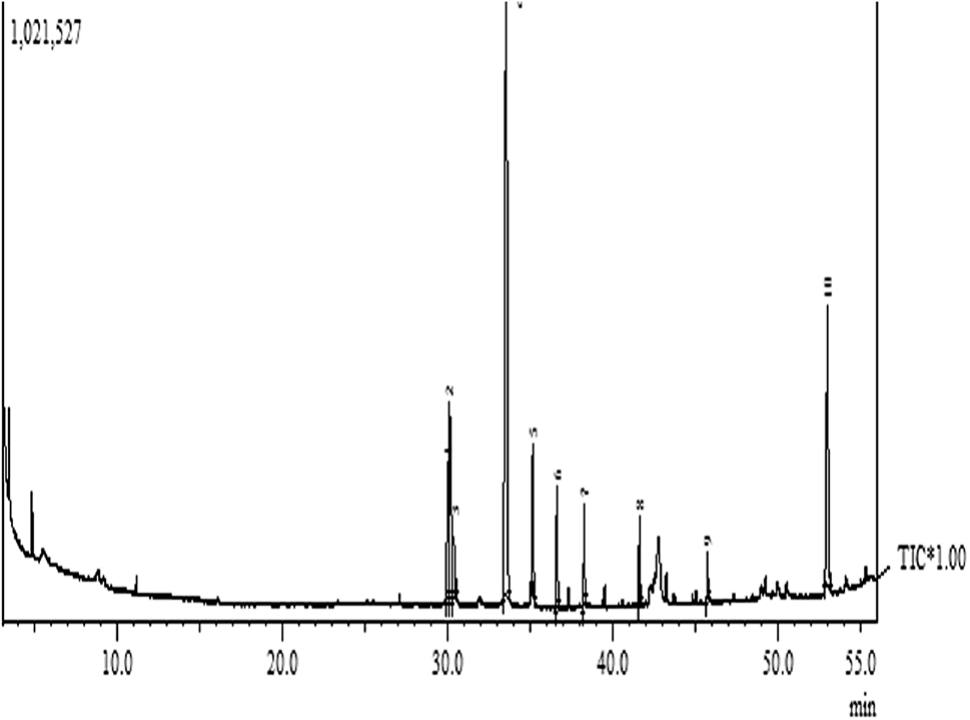
A typical GC–MS chromatogram of the ethanol extracts inhibition zone slices

GC–MS chromatogram analysis of the ethanol extract of inhibition zone parts *s*howed eight peaks that led to the identification of a number of compounds (Fig. 3). The various compounds were identified and characterized by comparing the mass spectra of the constituents with the NIST library (Table 1). Of the eight compounds identified in the inhibition zone, the prevailing compounds were dodecane (45.82 %), eicosane (15.65 %) and tridecane (15.62 %). Decane, tetradecane, pyrrolo [1,2-a] pyrazine-1,4-dione, hexahydro- and 2H-Chromene-3-carboxamide, 8-allyl-2-oxo-*N*-[2-(2-methyl-1H-indol-3-yl) ethyl]-found to be the minor components (4.792 %). Detected compounds and its derivatives showed various biological activities, for example, Ganendren et al. (2004) observed that the bis (quaternary phosphonium)-alkane 1,12-bis (tributylphosphonium) dodecane dibromide not only inhibited *cryptococcal* PLB1 but also exhibited in vitro antifungal activity. Eicosane and ethyl acetate extract of *Spirulena platensis* consisted of heptadecane and tetradecane showed antimicrobial activities against some Gram positive and Gram negative bacteria and *Candida albicans* (Ozdemir et al. 2004; Reid et al. 2005).

**Table 1:** GC-MS analysis of ethanol extract of ethanol extract of inhibition zone

No.	Compounds	Rt time	% Area	M.W.	Chemical formula
1	Decane	29.993	4.449	142.28	C_10_H_22_
2	Tridecane	30.117	15.62	184.36	C_13_H_28_
3	Dodecane	33.525	45.82	170.33	C_12_H_26_
4	Diethyl phthalate	35.175	7.830	222.23	C_12_H_14_O_4_
5	Tetradecane	36.625	4.792	198.38	C_14_H_30_
6	Pyrrolo[1,2-a]pyrazine-1,4-dione, hexahydro-	38.279	3.473	154.16	C_7_H_10_N_2_O_2_
7	2H-Chromene-3-carboxamide, 8-allyl-2-oxo-N-[2-(2-methyl-1H-indol-3-yl)ethyl]-	41.630	2.358	386.44	C_24_H_22_N_2_O_3_
8	Eicosane	53.024	15.65	282.54	C_20_H_42_

### Identification of the endophytic isolate

**Fig. 4:**
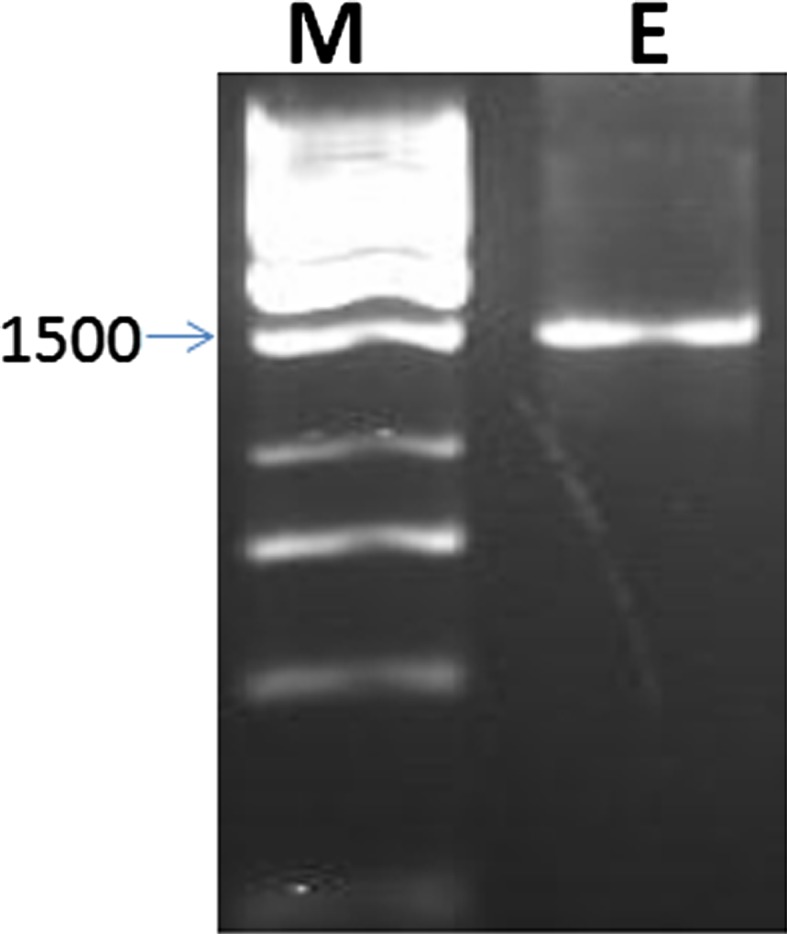
PCR of the 16S rRNA amplified gene, *M* 1 kb DNA ladder; *E* amplified gene product of the endophytic isolate

**Fig. 5:**
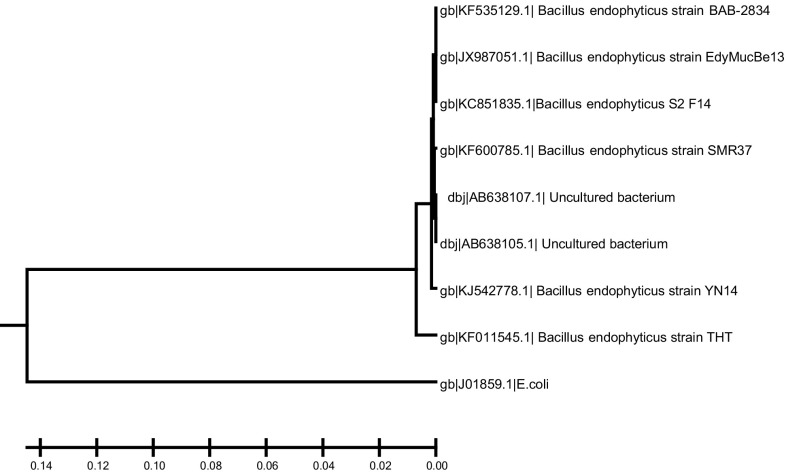
Phylogenetic tree of 16S rRNA gene of *B. endophyticus* isolate with related strains

Genomic DNA was extracted and the 16S rRNA gene was amplified using universal primers with 1500 pb product size (Fig. 4). The PCR amplified gene was purified and submitted for sequencing (Macrogen, Korea). The gene sequence revealed that the isolate is 99 % similar to *Bacillus endophyticus*. The sequence was deposited in GenBank with accession number KF011545. Multiple sequence alignment, molecular phylogeny and phylogenetic tree were performed using MEGA 5 software (Fig. 5).

### Unveiling of up-regulated or down-regulated genes

**Fig. 6:**
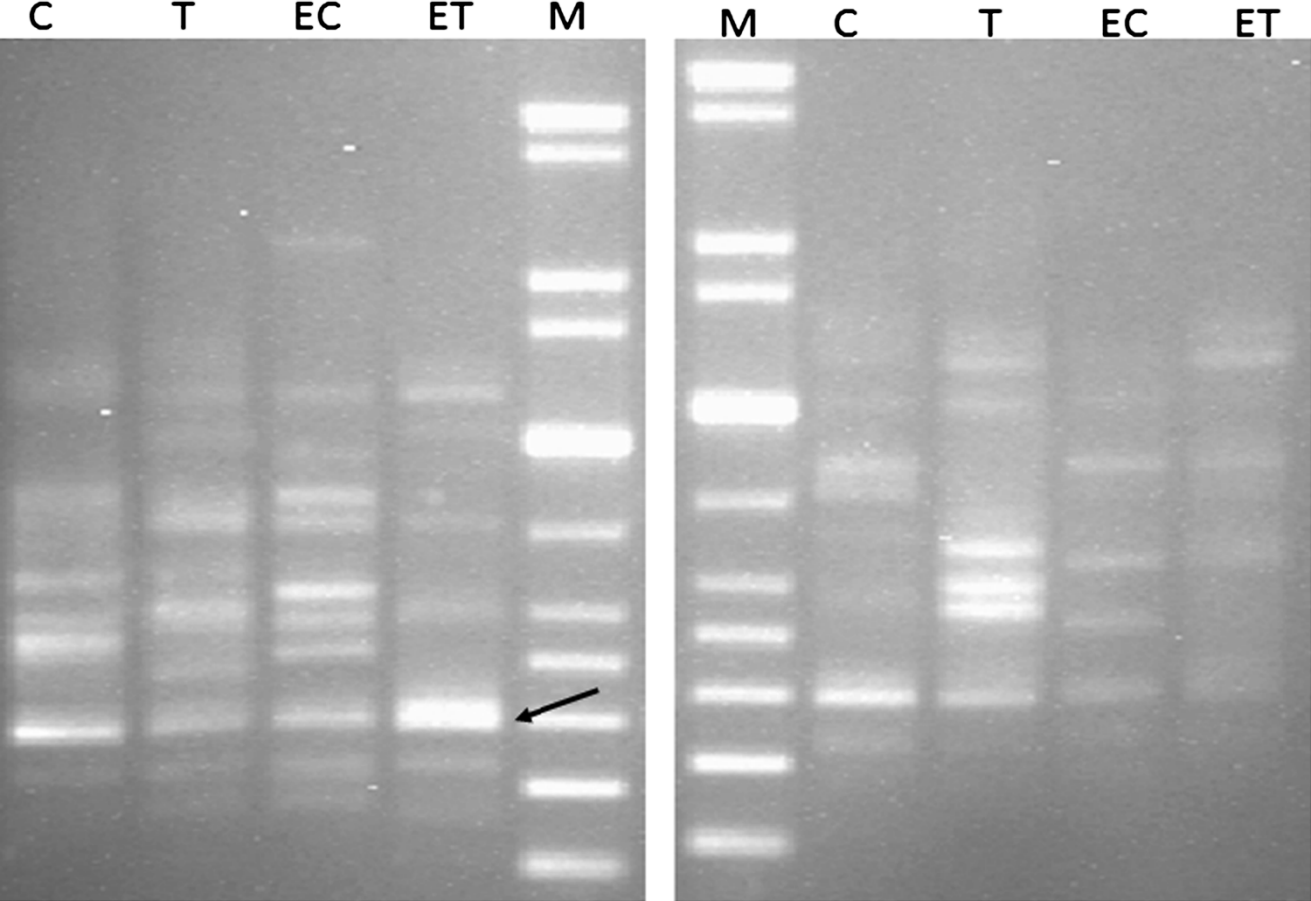
Differential display using Ea1 and Ea2 primers combined with OP1 reverse transcription primer. *M* 1 kb DNA ladder, *C* expressed genes of untreated *S. aureus*, *T* expressed genes of untreated *B. endophyticus*, *EC* expressed genes of *S. aureus* at the presence of *B. endophyticus*, *ET* expressed genes of *B. endophyticus* at the presence of *S. aureus*. The *black arrow* showed the up-regulated gene of *B. endophyticus* at the presence of *S. aureus*

Any external effect on the organism can affect its genetic behavior in a way of over or down expression of the genes according to the kind of the stimulant. The number and extent of the genes affected is not totally known to others. Differential display can offer some favors regarding this point, especially in the identification of some up-regulated or down-regulated genes under specific conditions. The discovery of these affecting genes is related to the sequence of the primers used during the reverse transcription process. We have used different primers that help to amplify different genes, whether over expressed or down expressed at the co-presence of antagonistic isolate and the pathogen. In our study, four up-regulated and down-regulated genes were chosen for gel purification and sequencing. However, only one of the sequenced genes was matched with genes deposited in GenBank. This gene was up-regulated, excised from the gel and submitted for gene sequencing (Fig. 6). The obtained sequence revealed that the concerned gene is serine protease-like gene. Serine protease is an enzyme that produced by many organisms including bacteria. It is widely produced by *Bacillus* species and found tremendous applications in pharmaceuticals (Bhunia et al. 2012 **)**.

Serine protease has been produced by many organisms, including plants, animals and microbes (Liu et al. 2010; Sakanari et al. 1989). Verma and Verma (2012) reported the isolation and purification of antimicrobial peptide from celomic fluid of Indian earthworm *Pheretima posthumous*. They identified the isolated peptide as serine protease that possesses a broad range of antimicrobial activity. The bacterial production and secretion of serine protease for antimicrobial function is not yet documented. However, we strongly propose that our isolate was able to produce serine protease for antimicrobial purpose. This claim is highly supported by the conditions which allow this enzyme not only to be produced but also to be over expressed. To our knowledge, this is the first study that illustrates the bacterial production of serine protease for antagonistic function against another genus of bacteria.

## Conclusion

The products of endophytic bacterial isolate *B. endophyticus* strain SA could be safely used as antibiotics alternative for treatment of methicillin resistant *S. aureus* (MRSA) strain.
